# Prevalence and Predictors of Pulmonary Tuberculosis among Prison Inmates in Sub-Saharan Africa: A Systematic Review and Meta-Analysis

**DOI:** 10.1155/2023/6226200

**Published:** 2023-05-22

**Authors:** Habtamu Belew Mera, Fasil Wagnew, Yibeltal Akelew, Zigale Hibstu, Sileshi Berihun, Workineh Tamir, Simegn Alemu, Yonas Lamore, Bewket Mesganaw, Adane Adugna, Tefsa Birlew Tsegaye

**Affiliations:** ^1^Department of Medical Laboratory Science, College of Health Sciences, Debre Markos University, Debre Markos 269, Ethiopia; ^2^Department of Pediatrics Nursing, College of Health Sciences, Debre Markos University, Debre Markos 269, Ethiopia; ^3^National Centre for Epidemiology and Population Health (NCEPH), College of Health and Medicine, The Australian National University, Canberra, Australia; ^4^Department of Public Health, College of Health Sciences, Injibara University, Injibara 40, Ethiopia; ^5^Department of Medical Laboratory Science, College of Health Sciences, Injibara University, Injibara 40, Ethiopia; ^6^Department of Public Health, College of Health Sciences, Debre Markos University, Debre Markos 269, Ethiopia; ^7^Department of Environmental Health Science, College of Health Sciences, Debre Markos University, Debre, Markos, 269, Ethiopia

## Abstract

**Introduction:**

Prisoners in Sub-Saharan Africa (SSA) are at a high risk of tuberculosis (TB) infection due to overcrowding and poor ventilation. Consequently, TB is a leading cause of morbidity and mortality in prison, and many inmates face a number of barriers to TB control and had limited information in the region. Thus, the aim of this systematic review and meta-analysis was to estimate the overall pooled prevalence of pulmonary TB and predictors among prison inmates in SSA.

**Methods:**

From 2006 to 2019, a systematic review and meta-analysis was conducted using various databases, including PubMed, Embase, Web of Science, and Scopus. The data were extracted in Microsoft Excel using a standardized data extraction format, and the analysis was carried out with STATA version 14. To detect heterogeneity across studies, the *I*^2^ and the Cochrane *Q* test statistics were computed. To determine the overall prevalence of TB and predictors among prison populations, a random effect meta-analysis model was used.

**Results:**

Of the 3,479 retrieved articles, 37studies comprising 72,844 inmates met the inclusion criteria. The pooled prevalence of pulmonary TB among prison inmates in SSA was 7.74% (95% CI: 6.46-8.47). In the subgroup analysis, the highest prevalence was found in the Democratic Republic Congo (DRC) (19.72%) followed by Zambia (11.68%) and then Ethiopia (9.22%). TB/HIV coinfection (OR 4.99 (95% CI: 2.60-9.58)), Body mass index (BMI < 18.5) (OR 3.62 (95% CI: 2.65-6.49)), incarceration (OR 4.52 (95% CI: 2.31-5.68)), and previous TB exposure (OR 2.43 (95% CI: 1.61-3.56)) had higher odds of pulmonary TB among inmates.

**Conclusion:**

The prevalence of pulmonary TB among SSA prison inmates was found to be high as compared to total population. TB/HIV coinfection, BMI, incarceration duration, and TB exposure were all predictors with pulmonary tuberculosis in prison inmates. As a result, emphasizing early screening for prisoners at risk of pulmonary TB is an important point to achieving global TB commitments in resource-limited settings.

## 1. Background

Tuberculosis (TB) is an airborne infectious disease mainly caused by *Mycobacterium tuberculosis* (MTB), which is still a major public health issue affecting person of all ages [1]. Adults in their most productive years are primarily affected, which accounted 88% [2, 3]. Globally, an estimated 10 million people developed TB disease in 2019, and there are 1.2 million TB deaths in HIV-negative people and an extra 208,000 among people living with HIV [4]. The major derivers of TB remain undernutrition, poverty, tobacco smoking, people infected with HIV, prisoners, household air pollutions, diabetes, and other comorbidities that impair the immune system which are all the risk of contracting TB [2–4]. TB affects 30% of the world's population and is the leading cause of death from a single infectious pathogen, accounting for 1.3 million deaths each year [5]. In 2020, the 30 high TB burden countries accounted for 86% of new TB cases, with 1.5 million people dying from the disease [6]. Low- and middle-income countries, including sub-Saharan Africa (SSA), account for 94% of all TB infections and deaths [2, 7]. In 2017, more than 10 million new cases of tuberculosis were reported worldwide, with one-third of these cases going unreported due to gaps in the healthcare system [7–9]. Although the World Health Organization (WHO) End Tuberculosis Global Strategy sets patronizing targets for 2020–2035, it aims to detect an estimated 90% of TB cases and reduce TB deaths by 95% in 2035 as compared to 2015, particularly among TB key populations who are most at risk of TB infection but have limited access to quality TB healthcare service [10–13]. To that end, WHO has prioritized the most vulnerable TB patients, including the poor, refugees, HIV-positive people, and prisoners, who are TB key populations [14]. Prison inmates are thought to be reservoirs for MTB transmission within their walls and in the community as a whole, but they are ignored for a variety of known reasons, including a lack of quality TB diagnostic services, overcrowding or stressful surroundings, a lack of perioding active case screening, comorbid illness, poor nutrition, and poor wall ventilation, particularly in SSA prisons [15–17]. Facts suggest that the risk of developing tuberculosis in prison is 6–30 times higher than in the general population, but 200 times higher in SSA, particularly in overcrowded prisons [13, 15]. In 2016, for example, a review of 24 SSA countries' prisons revealed TB prevalence ranging from 0.4 to 16.3% [18]. Another study published in the same year found that regional variation in TB prevalence was 5.3% in Southern and East Africa and 2.9% in Central and West Africa [18]. In SSA prisons, overcrowding and poor ventilation are severe, with data showing that 86% of countries with data had prison occupancy rates above 100%, increasing the risk of airborne TB infection [19]. Despite its burden, there is no aggregated data on pulmonary TB prevalence and its predictors in SSA prison inmates. As a result, the aim of this systematic review and meta-analysis was to precisely estimate the overall pooled prevalence and predictors of pulmonary tuberculosis among prison inmates in SSA.

## 2. Methods

### 2.1. Search Strategy and Study Identification

From 2006 to October 24, 2020, we systematically searched for articles published in international databases and electronic engines such as PubMed/MEDLINE, Cochrane Library, Google Scholar, Embase, Scopus, and Web of Science. In addition, we searched the reference lists of the included studies for relevant articles. The grey literatures were found by communicating and searching the digital/repository library, the institution's official website, colleagues, researchers, and other scholars. To search relevant articles for this study, the following MeSH terms and keywords were used: “prevalence”/“magnitude”/“proportion”, “tuberculosis”, “pulmonary tuberculosis”, “PTB”, “associated factors”/“predictors”/“risk factors”, “prison inmates”/“prisoners”, “Sub-Saharan Africa”, and “SSA”. The MeSH terms were used separately and/or in combination using Boolean operators like “OR” or “AND” (*see Supplementary file*1). The literature search for the aforementioned databases was done from June 01, 2020, to October 24, 2020. The review protocol was entered into the International Prospective Register of Systematic Reviews (PROSPERO), which is maintained by the University of York Centre for Reviews and Dissemination (CRD4202016764). Preferred Reporting Items for Systematic Reviews and Meta-Analysis (PRISMA) guidelines were followed in this study [20, 21].

### 2.2. Eligibility Criteria

#### 2.2.1. Inclusion Criteria

The contents of each included article were independently reviewed by the two investigators (HB and TB). When the specimen for laboratory processing is sputum, the prevalence of pulmonary TB was reported, and quality assurance measures for laboratory diagnostic methods (volume of sputum, acid-fast bacilli (AFB), fluorescence microscopy (FM), culture, and GeneXpert MTB/RIF), diagnostic technique of standard operating procedures, and methodological quality of each original study were carefully examined.

*Study area*: all novel or primary studies conducted in SSA prisons were taken into account.

*Publication condition*: from January 1, 2006, to October 24, 2020, articles published in peer-reviewed journals were included.

*Study design*: observational study designs with original articles contain data reporting the prevalence or number of confirmed TB cases and predictors of pulmonary TB among SSA prison inmates.

*Language*: only English language publications were considered.

#### 2.2.2. Exclusion Criteria

After reviewing the abstracts and full texts of the papers, the two independent reviewers (HB and TB) extracted data with care. We excluded incomplete data, full-text inaccessible articles, studies conducted outside of SSA after contacting the primary authors via email or phone, articles that did not report the point prevalence, reviewed articles, and studies conducted in the general population.

### 2.3. Data extraction

The three authors (HB, TB, and ZH) extracted the necessary data using a standardized data extraction format in an excel spreadsheet. The following information was extracted from the studies: first author, country of study, diagnostic methods, specimen type, study design, year of publication, sample size, area of prisoners, and tuberculosis prevalence. When the three authors disagreed, a fourth author (FW) was consulted, and disagreements were resolved through consensus and discussion.

### 2.4. Quality Assessment Tool

To assess the quality of the studies, the two authors (HB and TB) used the Newcastle-Ottawa quality assessment tool, which was modified for nonrandomized and cross-sectional studies [22, 23]. Quality assessment tool was modified for nonrandomized and cross-sectional studies. There are three indicators in the tool. The first section was given five stars and assessed the quality of the study's methodology; the investigators also evaluated the quality of laboratory diagnostic procedures. The second section graded the studies' comparability out of three stars. The third section graded the quality of the original articles based on their statistical analyses, which were graded out of two stars. Studies with medium (50% of quality assessment criteria was met) and high quality (6 out of 10 scales) were included in the analysis.

### 2.5. Statistical Data Analysis

The necessary data was extracted using Microsoft Excel and analyzed using STATA/SE version 14. Tables were used to summarize the included articles, and the forest plot was used to estimate the study effect size and confidence interval. Using the binomial distribution formula, the researchers calculated the standard error of TB prevalence for each original article. Using the Cochrane *Q* statistics and *I*^2^ test, we examined heterogeneity in the reported prevalence of pulmonary TB [24]. Heterogeneity is classified as high with >75%, substantial with 50-75%, moderate with greater than 25 and less than 50%, and low with 25% for *I*^2^ [25]. To determine the pooled effect of the original studies, a random effects model with Der Simonian and Laird's statistical methods was used. To identify potential sources of heterogeneity, a univariate metaregression analysis was performed using the publication year, sample size, and confirmed TB. Furthermore, subgroup analysis was performed in these studies using variables to reduce the random heterogeneity between the estimates of the original studies [26]. Potential publication bias was evaluated subjectively using a funnel plot and objectively using Egger's weighted correlation and Begg's regression cutoff tests at a 5% significance level; a *p* value of 0.05 indicates the presence of publication bias [27]. Another subjective assessment of publication bias is a funnel plot, which is plotted by effect size per study versus standard error of effect size. Each dot represents a single study, and symmetric dots with an inverted funnel shape indicate that there is no publication bias. If the random effects model detects publication bias, the estimate is determined using the trim and fill analysis.

## 3. Results

### 3.1. PRISMA Flow Chart

**Figure 1:**
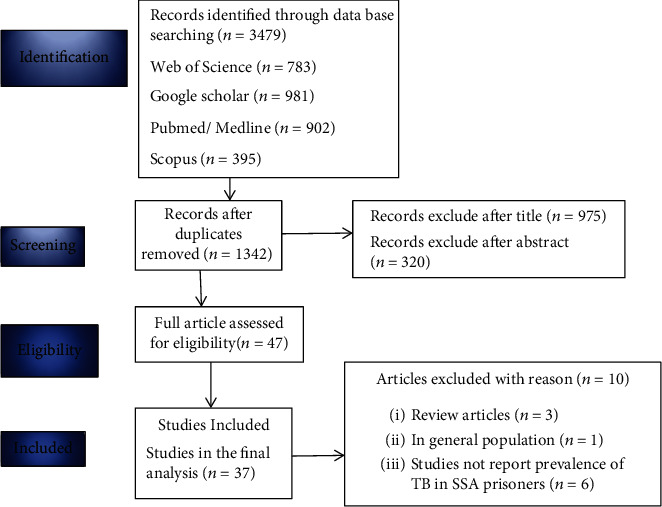
PRISMA flow chart selection process for the systematic review and meta-analysis of the prevalence of pulmonary tuberculosis and predictors among prison inmates in SSA, 2020.

The authors extensively extracted a total of 3,479 articles from international databases and other electronic engines. Because of duplications, 2131 records were removed, and 975 and 320 records were excluded from the remaining 1,342 articles after reviewing their titles and abstracts, respectively. Forty-seven articles were fully evaluated for inclusion and exclusion based on the study eligibility criteria. We removed 10 articles due to population and outcome of interest differences such as Indonesia [28], sub-Saharan Africa [5, 29], Ethiopia [30], Malaysia [9], Nepal [31], Brazil [32], Southwest Iran [33], Portugal [34], and Uganda [35]. Finally, 37 studies with a total of 72,844 prisoners met the eligibility criteria and were included in the final systematic review and meta-analysis (Figure 1).

### 3.2. Explanation of the Original Studies

The following are the characteristics of the 37 original studies included in this review. These studies were published between 2006 and 2019, and the current study included 11 country prison inmates. The majority of the studies, 14/37 (37.8%), were conducted in various regions of Ethiopia [36–49]: four in Nigeria [50–53], four in South Africa [54–57], two in Malawi [58, 59], two in Uganda [60, 61], three in Zambia [62, 63], two in Cameroon [64, 65], one in Côte d'Ivoire [66], two in Ghana [67, 68], one in Tanzania [69], and two in Democratic Republic Congo (DRC) [70, 71], whereas WHO has classified five of them among the thirty high burden country lists for TB, TB/HIV, and MDR-TB [8]. The highest prevalence of pulmonary TB among prison inmates (23.08%) was reported in Nigeria from Aba Federal prison [51], while the lowest prevalence (1.42%) of pulmonary TB was reported among eighteen prisons in Malawi [58]. In this meta-analysis, 72,844 prison inmates were included in SSA prisons to evaluate the pooled prevalence of pulmonary TB and its predictors. Concerning the study design, almost all 89.2% (33/37) of the studies were cross-sectional study. Study-specific sample size ranged from 52 in Nigeria [51] to 31,547 in South Africa [57]. All the original studies used sputum as a specimen for diagnosis of TB, and different diagnostic techniques were carried out to confirm pulmonary TB in the prisoners such as AFB or direct light microscopy, fluorescent microscopy (FM), culture, and GeneXpert. The quality score of all the 37 studies ranged six to nine (Table 1).

### 3.3. The Quality Score of the Original Studies

All of the 37 studies were undergoing the quality assessment of methodological, and methods of laboratory diagnostic criteria were critically justified by using the Newcastle-Ottawa quality assessment tool [22]. Regarding the sampling technique, almost all of the included studies used active case finding approach ≥ two or less duration of cough during the study period. Presumptive symptom-based tuberculosis screening using standardized tuberculosis screening protocols was used. All the included studies used different laboratory diagnostic techniques to confirm pulmonary tuberculosis in the prisons (Table 2).

### 3.4. Meta-Analysis (Heterogeneity, Publication Bias, and Sensitivity Analysis)

**Figure 2:**
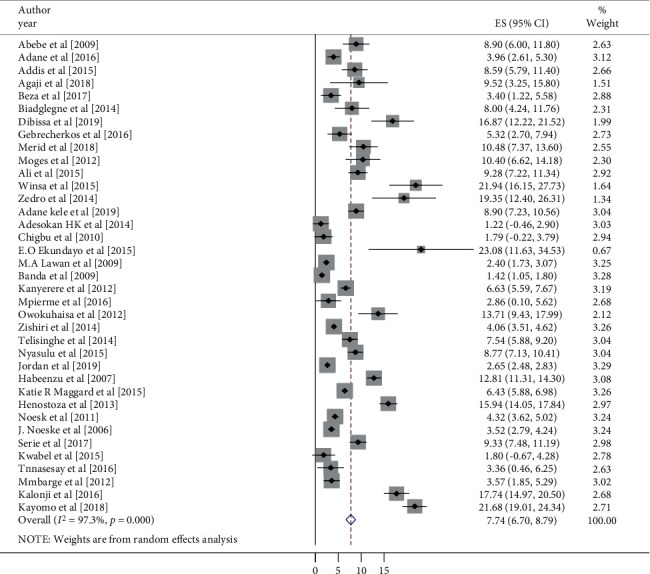
The forest plot of the pooled prevalence of pulmonary tuberculosis among prison inmates is SSA.

The overall pooled prevalence of pulmonary TB among SSA prison inmates in the random effects model was 7.74% (95% CI, 6.70-8.79, *I*^2^ = 97.3%) (Figure 2). The pooled point incidence rate in this meta-analysis from the thirty-seven studies per 100,000 prison inmates was estimated to be 10,700 per 100,000 prison inmates (95% CI: 10,340–10,952). The results of the included studies' sensitivity analyses revealed that no study significantly influenced the results, with effect sizes ranging from 2.82 to 3.07. Different factors potentially associated with heterogeneity, such as sample size, publication year, and confirmed pulmonary TB, were checked by advanced metaregression model using univariate metaregression to identify the possible source of heterogeneity, and all potential variables of TB prevalence were not statistically significant (Table 3).

### 3.5. Subgroup Analysis

A subgroup analysis was performed to investigate the source of heterogeneity based on country, sample size, and study publication year. According to this, the DRC has the highest prevalence of pulmonary TB, with a prevalence of 19.72% (95% CI: 15.86-23.59) followed by Zambia 11.68% (95% CI: 5.61-17.75), Côte d'Ivoire 9.33% (95% CI: 7.46-11.19), and Ethiopia 9.22% (95% CI: 6.59-11.85). With respect to the sample size, the prevalence of pulmonary TB in prisoners was higher 8.5% (95% CI: 6.00-11.01) in studies having a sample size of <384 as compared to those participants which have a sample size of ≥384 who have 7.3% (95% CI: 6.07-8.52). Concerning years of publication, the prevalence of pulmonary TB among prison inmates in SSA was higher in studies which have been conducted after 2015, 9.01% (95% CI: 7.10-10.92), as compared to studies which had been carried out before 2015, 6.72% (95% CI: 5.16-8.28) (Table 4).

**Figure 3:**
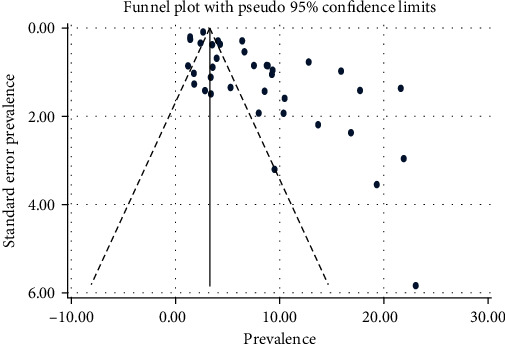
The funnel plot of the meta-analysis containing the 37 studies.

In this review, we observed that no publication bias was statistically significant between the primary studies by observing the funnel plot, Begg's (*p* value = 0.147), and Egger's regression asymmetry test (*p* value = 0.143) (Figure 3).

### 3.6. Predictors of Pulmonary Tuberculosis among Prison Inmates

**Figure 4:**
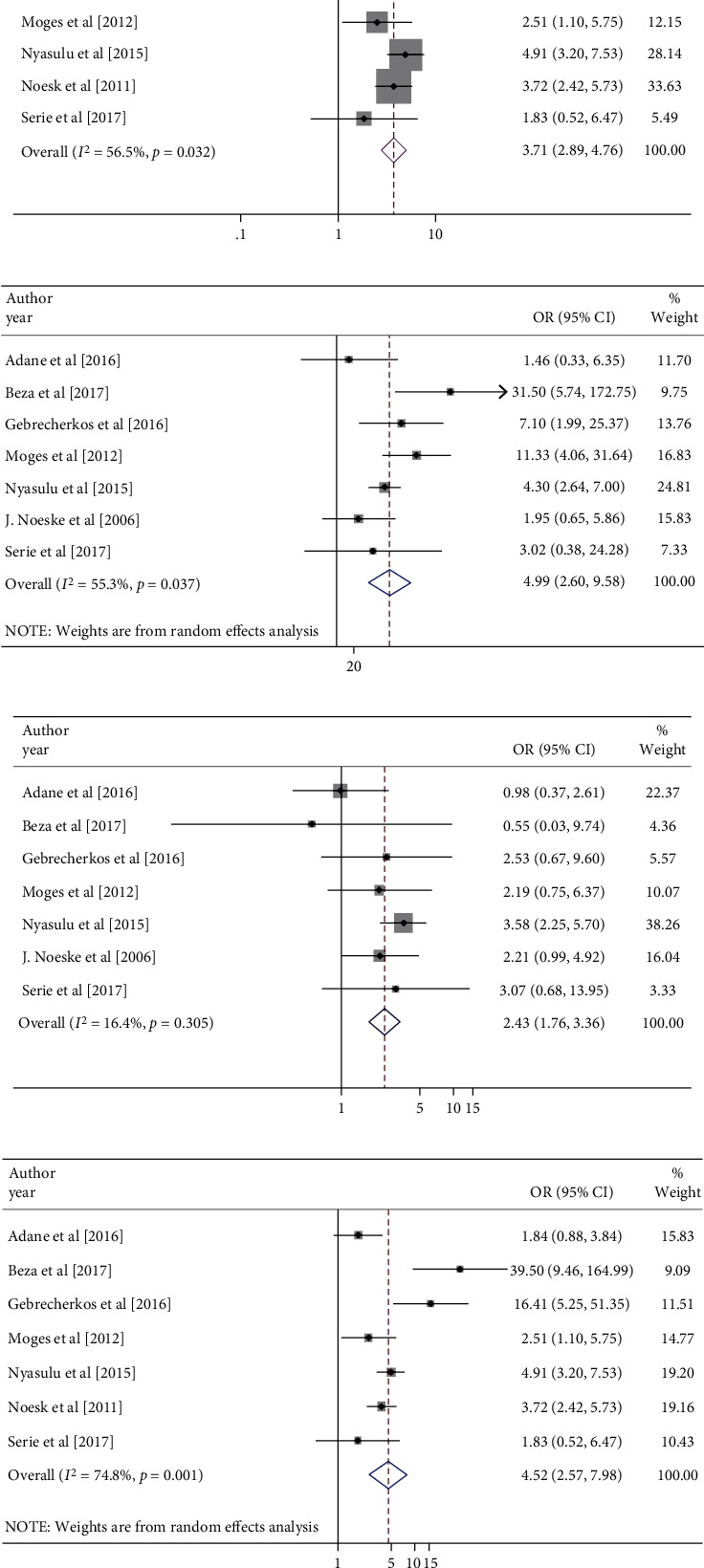
(a) Forest plot illustrating BMI associated with pulmonary TB among prison inmates, in SSA. OR: odds ratio. (b) Forest plot illustrating previous TB/HIV coinfection associated with pulmonary TB among prison inmates, in SSA. OR: odds ratio. (c) Forest plots showing pooled odds ratio of previous TB history associated with pulmonary tuberculosis among prison inmates in SSA. OR: odds ratio. (d) Forest plots illustrating pooled odds ratio of duration of incarceration associated with pulmonary tuberculosis among prison inmates in SSA. OR: odds ratio.

Only seven of the 37 studies had data that could be analyzed to determine the associations [37, 40, 43, 45, 56, 65, 66]. Body mass index (BMI), TB/HIV coinfection, a history of TB, and incarceration, which prison inmates live in the prison ≥ 6 months, were identified as potential predictors for pulmonary TB among prison inmates in SSA countries (Figure 4). BMI of 18.5 kg/m^2^ was found to be significantly associated with pulmonary tuberculosis in prison inmates adjusted odds ratio (AOR): 3.62 (95% CI: 2.31-5.68). Prison inmates who have a body mass index of <18.5 kg/m^2^were 3.62 times more likely to have pulmonary TB as compared to those prisoners who have a BMI of ≥18.5 kg/m^2^ (Figure 4(a)). When compared to their counterparts, inmates with HIV were 4.99 times more likely to be infected with pulmonary tuberculosis, AOR: 4.99 (95% CI: 2.60-9.58) (Figure 4(b)). Similarly, a meta-analysis of this study found that prison inmates with a history of TB were more likely to contract pulmonary TB (Figure 4(c)). The pooled estimate of the seven studies also revealed that long duration of incarceration (≥6 months) was strongly related to pulmonary tuberculosis among prison inmates, AOR: 4.52 (95% CI: 2.57, 7.98) (Figure 4(d)).

## 4. Discussion

Tuberculosis remains a major public health threat worldwide, particularly in the SSA region. The current meta-analysis found a high prevalence of pulmonary TB among SSA prison inmates. TB/HIV coinfection, BMI, long duration of incarceration, and previous TB exposure were predictors of pulmonary TB infection among prison inmates. The current meta-analysis estimated that the overall pooled prevalence of TB among prison inmates using the 37 studies was 7.74%. This prevalence is similar with the study conducted in Malaysia 7.7% [9], Southwest Iran 7.9% [33], and SSA (6.4%-8.8%) [30, 38, 41, 56, 63] as previously reported studies among prison inmates. However, the finding of this meta-analysis was higher than studies in South Thailand (2.1%) [74], Peru (2.51%) [75], Asian countries (4.5%) [76], Brazil [77], and SSA (2%-3.6%) [37, 55, 63] as reported in a previous systematic review conducted from prison inmates. Moreover, the finding of our study was lower than a study conducted in Brazil diagnosed with smear, GeneXpert, and culture 12%-12.9% [78, 79], Nepal sputum smear and GeneXpert test 10% [31], and SSA 10%-13.7% [44, 61] as conducted in previous reports among prison inmates. The possible explanations for the observed variations might be attributable to the difference in the geographical variation, overcrowding, method of diagnosis, and number of prisoners in a cell with poor ventilation. In this systematic review and meta-analysis, the pooled estimated incidence of pulmonary TB among prisoners was 10,700/100,000, which is higher than WHO target for end TB strategy incidence in 2019, which is approximately130/100,000 [8]. Despite this, high incidence of TB in prisons could be possibly the fact that they are a forgotten population in the case of early screening, diagnosis, and treatment of TB in a high-risk population like prison inmates.

The subgroup analysis of this meta-analysis also showed that the prevalence of pulmonary TB was higher in prisoners incarcerated in DRC 19.72%, Zambia 11.68%, and Ethiopia 9.22% as compared to other countries in SSA. This difference in the prevalence of pulmonary TB within countries in the prisons might be the difference in the diagnostic technique, screening method, overcrowded, and sociocultural and socioeconomic variations of the study participants. Concerning to study year, the prevalence of pulmonary TB was significantly higher than in those studies conducted after 2015 as compared to those studied before 2015. This difference might be hypothesized that due to implementation variations in strategy targets and milestones for the underlying in the end TB strategy in SSA. This might be integrated, patient-centered care and prevention for high-risk populations of TB, bold policies and supportive systems, high-level political commitment and shortage of enhanced resources, and intensified research and innovations to end TB epidemic in high-risk groups and new diagnostics tools, drugs, and vaccines in the SSA region were needed [14].

This meta-analysis examined that the predictors of BMI, presence of TB/HIV coinfection, previous TB history, and duration of incarceration were meta-analyzable and were significantly associated with the prevalence of pulmonary TB among prison inmates in SSA. Prison inmates who are underweight were 3.62 more likely to be infected by pulmonary TB than those prison inmates who had a BMI of ≥18.5 kg/m^2^. This finding is similar with a study reported by Valença et al. and Casha and Scarci [79, 80]. The possible explanation for this is that a low BMI in body construct might be in some way predisposed to TB reactivation in the lung due to congenital apical lung bullae and biomechanical pleural stress that increases in apical pleural stress in low anteroposterior diameter chests on coughing. Underweight impairs the immune system; these might alter cytokine synthesis, reduce antigen response, and diminish function of natural killer cells, dendritic cells, and macrophages, thus might affect TB incidence. The presence of previous TB/HIV coinfection of the prison inmates was significantly associated with the expansion of pulmonary TB. Prisoners with previous TB/HIV coinfection were 4.99 times more likely to have active pulmonary TB than prisoners who had not TB/HIV infection before. This is because people living with HIV are more likely infected by *Mycobacteria tuberculosis* where HIV weakens the immune system due to the depletion of CD_4_T cells and HIV upregulates *M. tuberculosis* entry into receptors on macrophages [1, 81] that weakens the body to fight the bacteria and exerts immense burden in healthcare systems in resource liming settings like SSA. Similarly, the results of this meta-analysis showed that prisoners that have not previously TB history have protective associations with enhancement of pulmonary TB. Prison inmates who have presence of previous TB history/contact were 2.43 times more likely to develop pulmonary TB as compared to its counterparts. The finding of this study is consistent with a study conducted in South India which is 3.64 times more infected [82]. Long duration of imprisonment of prisoners was 4.52 times more likely infected by *M. tuberculosis* as compared to those prisoners whose duration of incarceration is short [50, 56, 82]. This might be because prisoners are not closed system, due to the number of people entering, departing, and reentering and poor environmental situation possibly increasing the transmission probability [83].

## 5. Limitation of the Study

Despite the fact that the authors conducted a comprehensive search using various databases for articles on the prevalence and predictors of pulmonary TB among prison inmates in SSA, this systematic review and meta-analysis failed to include reports published in languages other than English. We found studies in only eleven SSA countries, with other countries underrepresented due to the small number of studies included in prisons. Furthermore, studies and important data published in peer-reviewed journals between 2006 and 2019 may have gone overlooked.

## 6. Conclusion

In this systematic review and meta-analysis, the pooled prevalence of pulmonary TB among prisoners in SSA was considerably high which needs special attention to attain the end TB strategy. Underweight, previous TB/HIV coinfection, long duration of incarceration, and previous TB exposure/contact history are predictors of pulmonary TB infection among prisoners in SSA. Therefore, establishing systems for providing adequate nutrition and balanced diet, early screening, diagnosis of long imprisonment, treatment of TB/HIV coinfection, and contact history should be implemented to address this problem and succeed the control and end TB strategy in the SSA.

## Figures and Tables

**Table 1:** Descriptive summary of 37 studies reporting the prevalence and associated factors of pulmonary tuberculosis among prison inmates included in the systematic review and meta-analysis in sub-Saharan Africa, 2020 (*n* = 72,844).

Author (year)	Country	Study design	Method of diagnosis	Specimen type	Sample size	QS (10pts)	Prevalence with 95% CI
Abebe et al. (2011) [36]	Ethiopia	C-S	AFB, culture	Sputum	371	7	2.07 (2.6, 5.5)
Adane et al. (2016) [37]	Ethiopia	C-S	AFB, culture	Sputum	809	7	3.45 (2.8, 10.7)
Addis et al. (2015) [38]	Ethiopia	C-S	AFB, culture	Sputum	384	7	4.9 (1, 18)
Agajie et al. (2018) [39]	Ethiopia	C-S	AFB	Sputum	84	8	11 (10, 29)
Gizachew Beza et al. (2017) [40]	Ethiopia	C-S	GeneXpert	Sputum	265	7	8.4 (8, 15)
Biadglegne et al. (2014) [41]	Ethiopia	C-S	GeneXpert	Sputum	200	7	7 (5, 21)
Dibissa et al. (2019) [42]	Ethiopia	C-S	AFB, culture, and GeneXpert	Sputum	249	8	6 (5, 28)
Gebrecherkos et al. (2016) [43]	Ethiopia	C-S	FM, GeneXpert	Sputum	282	7	6.04 (5, 16)
Merid et al. (2018) [44]	Ethiopia	C-S	AFB, GeneXpert	Sputum	372	9	5 (1, 20)
Moges et al. (2012) [45]	Ethiopia	C-S	FM	Sputum	250	7	6 (1, 22)
Mohammed (2017) [46]	Ethiopia	C-S	AFB, culture	Sputum	765	9	3 (2, 16)
Winsa and Mohammed (2015) [47]	Ethiopia	C-S	AFB	Sputum	196	7	6 (2, 34)
Zedro et al. (2014) [48]	Ethiopia	C-S	AFB, culture	Sputum	124	8	8 (3, 35)
Adane et al. (2019) [49]	Ethiopia	C-S	AFB	Sputum	1124	9	3 (1, 14)
Adesokan et al. (2014) [50]	Nigeria	C-S	Culture	Sputum	164	9	7.7 (5, 16)
Chigbu and Iroegbu (2010) [53]	Nigeria	C-S	Culture	Sputum	168	9	13 (7, 16.7)
Ekundayo et al. (2015) [51]	Nigeria	C-S	AFB	Sputum	52	9	12 (2, 46)
Lawal et al. (2009) [52]	Nigeria	C-S	AFB	Sputum	2002	8	2 (1, 6.7)
Banda et al. (2009) [58]	Malawi	Retrospective	AFB	Sputum	3794	9	2 (1.7, 4.6)
Kanyerere et al. (2012) [59]	Malawi	C-S	AFB	Sputum	2217	8	2 (0.5, 10)
Mpeirwe et al. (2016) [60]	Uganda	C-S	FM, culture	Sputum	140	8	8 (6, 5.19)
Owokuhaisa et al. (2014) [61]	Uganda	C-S	AFB	Sputum	248	7	6 (2, 25)
Zishiri et al. (2015) [55]	S.A	C-S	GeneXpert	Sputum	4945	9	1.4 (1, 6)
Telisinghe et al. (2014) [54]	S.A	C-S	AFB, culture	Sputum	968	8	3 (1, 14)
Nyasulu et al. (2015) [56]	S.A	C-S	AFB, FM	Sputum	1140	7	3.5 (1, 15)
Jordan et al. (2019) [57]	S.A	C-S	GeneXpert	Sputum	31547	8	0.6 (0.2, 4)
Habeenzu et al. (2007) [72]	Zambia	Case finding	AFB, FM, and culture	Sputum	1921	8	8 (2, 17)
Maggard et al. (2015) [63]	Zambia	C-S	FM	Sputum	7638	8	4 (3.1, 9)
Henostroza et al. (2013) [62]	Zambia	C-S	FM	Sputum	1430	7	2.4 (1, 21)
Noeske et al. (2011) [65]	Cameroon	Case finding	AFB, culture	Sputum	3219	8	1.7 (0.9, 7.7)
Noeske et al. (2006) [64]	Cameroon	C-S	AFB, culture	Sputum	2474	7	2 (0.3, 7.4)
Séri et al. (2017) [66]	Côte d'Ivoire	C-S	AFB, culture	Sputum	943	9	3 (1, 15)
Kwabla et al. (2015) [67]	Ghana	C-S	AFB	Sputum	111	7	9 (7, 20)
Sesay (2016) [68]	Ghana	C-S	GeneXpert	Sputum	149	7	8 (5, 19)
Mmbaga (2013) [69]	Tanzania	C-S	AFB	Sputum	448	8	5 (3.9, 13)
Kalonji et al. (2016) [70]	DRC	C-S	AFB, FM	Sputum	733	9	3 (1, 24.3)
Kayomo et al. (2018) [71]	DRC	C-S	GeneXpert	Sputum	918	6	2.9 (1, 27.4)

Key: AFB: acid-fast bacilli; FM: fluorescence microscopy; C-S: cross-sectional; S.A: South Africa; DRC: Democratic Republic Congo; SNNPR: Southern Nations, Nationalities, and People's Region; QS: quality score.

**Table 2:** Quality assessment techniques of the primary studies for the prevalence and predictors of pulmonary tuberculosis among prison inmates in SSA, 2020.

Sampling techniques for screening of TB in the prison for the selection of participants	Mass screening in the prison (census)	References
		[36, 38, 39, 41, 42, 43, 51, 58, 62, 63, 65, 67, 68, 70, 71, 73]
	Consecutive convenient sampling	[40, 44, 48, 49, 53, 59]
	Random sampling technique	[37, 50, 54–57, 62, 64, 66, 69]

Diagnosis methods: bacteriological confirmation of pulmonary tuberculosis in the prison inmates	Direct light (AFB)/FM and culture (Lowenstein-Jensen)	[48, 53, 54, 60, 64–66, 72, 73]
	GeneXpert MTB/RIF	[39, 40, 42, 55]
	Direct light microscopy(AFB)	[38, 47, 51, 58, 59, 61, 67, 69, 70]
	Direct microscopy, GeneXpert MTB/RIF, and culture	[41]
	Digital chest X-ray, direct microscopy, and culture	[62, 66]
	Direct microscopy and chest X-ray	[52, 68]
	Digital chest X-ray, GeneXpert MTB/RIF, and direct microscopy	[49, 57]
	Direct microscopy/FM and GeneXpert	[43, 44]
	Culture (Lowenstein-Jensen)	[36, 37, 50]
	GeneXpert and culture	[71]
	FM	[45, 56, 63]

Key: AFB: acid-fast bacilli; FM: fluorescent microscopy.

**Table 3:** The predictors of pulmonary TB among prison inmate in SSA in the current metaregression model based on univariate metaregression, 2020.

Variables	Coefficient	*p* value
Sample size	-0.0013	0.148
Publication year	0.406	0.172
Confirmed pulmonary tuberculosis	-0.0006	0.992

**Table 4:** Subgroup analysis of the prevalence of pulmonary tuberculosis among prison inmates in SSA, 2020.

Variables	Characteristics	Included studies	Sample size	Prevalence with 95% CI
Country	Ethiopia	14	5,475	9.22 (6.59, 11.85)
	Nigeria	4	2,386	2.34 (0.40, 4.58)
	Malawi	2	6,011	4.00 (1.1, 9.11)
	Uganda	2	388	8.15 (2.47, 18.79)
	South Africa	4	38,600	5.56 (3.61, 7.50)
	Zambia	3	10,989	11.68 (5.61, 17.75)
	Cameroon	2	2,386	3.92 (3.14, 4.71)
	Côte d'Ivoire	1	943	9.33 (7.46, 11.19)
	Ghana	2	260	2.46 (0.58, 4.34)
	Tanzania	1	448	3.57 (1.85, 5.29)
	DRC	2	1,651	19.72 (15.86, 23.59)

Sample size	<384	17	3,425	8.5 (6.00, 11.01)
	≥384	20	69,419	7.3 (6.07, 8.52)

Publication year	<2015	17	24,943	6.72 (5.16, 8.28)
	≥2015	20	47,901	9.01 (7.10, 10.92)

## Data Availability

The literature analyzed in the current study is available from the online data sources by using the reference listed or available from the corresponding author on a reasonable request.

## Abbreviations

AFB: Acid-fast bacilli
AOR: Adjusted odds ratio
BMI: Body mass index
CI: Confidence interval
C-S: Cross-sectional
FM: Fluorescent microscopy
HIV: Human immunodeficiency virus
MTB: *Mycobacterium tuberculosis*
OR: Odds ratio
PRISAM: Preferred Reporting Items for Systematic Review and Meta-Analysis
SSA: Sub-Saharan Africa
TB: Tuberculosis
WHO: World Health Organization.

## Supplementary Materials

Supplementary Materials Supplementary file 1: searching approach for different databases.
