# Supplementary material for: Prevalence and Predictors of Pulmonary Tuberculosis among Prison Inmates in Sub-Saharan Africa: A Systematic Review and Meta-Analysis
Source: Tuberc Res Treat. 2023 May 22;2023:6226200. doi: 10.1155/2023/6226200 (PMC10228229; doi:10.1155/2023/6226200)
Supplement: Supplementary Materials — Supplementary file 1: searching approach for different databases. [file 6226200.f1.docx]

Supplementary files

Searching approach for different databases

| Search strategy. | Database collection keywords or descriptors |
| --- | --- |
| PubMed/Medeline | (("Mycobacteria tuberculosis "[Supplementary Concept] OR " Mycobacteria tuberculosis"[All Fields] OR "MTB"[All Fields] OR "MTB"[Title] OR "PTB"[Title] OR "Pulmonary Tuberculosis infection"[Title/Abstract] AND "prison inimates"[Title/Abstract]) OR "prisoners"[Title/Abstract] AND "sub sahran Africa"[Title/Abstract]) OR (sub sahran Africa"[Title/Abstract] |
| Scopus | (Pulmonary AND PTB AND infection OR Pulmonary AND PTB OR lung diseases AND *Mycobacterium tuberculosis* OR Pulmonary Tuberculosis) |
| Cochrane Library | (title:(PTB) OR abstract:(PTB)) OR (title:(Mycobacterium tuberculosis) OR abstract:( *Mycobacterium tuberculosis*)) OR (title:(Pulomonary Tuberculosis) OR abstract:(PTB Diseases)) OR abstract:(associated factors)) OR (title:(magnitude) OR abstract:(lung diseases)) OR (title:(Prevalence) AND prison inmate title:( SSA)) |
| Web of Science | TITLE:(PrevalenceOR Magnitude OR pulmonary tuberculosis OR Associated factor AND prisoners AND Sub-Saharan Africa |
| EMBASE | 'Magnitude of PTB OR Prevalence of PTB OR Lung diseases OR Pulmonary tuberculosis infections OR Risk Factors AND prison inmate AND sub-saharan Africa' |
